# Biomechanical interactions of endodontically treated tooth implant-supported prosthesis under fatigue test with acoustic emission monitoring

**DOI:** 10.1186/s12938-016-0140-y

**Published:** 2016-02-24

**Authors:** Shao-Fu Huang, Wan-Rung Chen, Chun-Li Lin

**Affiliations:** Department of Biomedical Engineering, National Yang-Ming University, 2 No.155, Sec.2, Linong Street, 112, Taipei, Taiwan

**Keywords:** Endodontically treated tooth, Dental implant, Splinting, Biomechanics, Acoustic emission, Fatigue

## Abstract

**Background:**

This study investigated the biomechanical interactions in endodontically treated tooth implant-supported prosthesis (TISP) with implant system variations under dynamic cyclic loads monitored using the acoustic emission (AE) technique.

**Methods:**

Macrostructure implants using a taper integrated screw-in (TIS; 2-piece implant) and a retaining-screw (RS; 3-piece implant) connected to an abutment were used for this investigation and their corresponding mechanical resistances in conformity with the ISO 14801 standard were evaluated. The endodontically treated TISP samples were constructed containing TIS and RS implants splinted to the second premolar with fatigue tests performed by applying occlusal force onto the premolar simulating the bending moment effect. The numbers of accumulated AE signals in the fatigue tests and failure modes for the sample were recorded to evaluate the mechanical resistance.

**Result:**

The maximum load in the static test for RS (3-piece) implant (797N) was significantly higher than that for the TIS (2-piece) implant (559N). Large deformations were found at abutment screws in both RS and TIS implants. Although the numbers of accumulated AE signals for the TIS implant (72511) were higher than those for the RS implant (437), statistical non-significant differences were found between TIS and RS implants. No obvious damage was noted in endodontically treated TISP samples using RS implants but two of the corresponding TIS implants fractured in the abutment screws.

**Conclusions:**

Splints with RS (3-piece) implant prosthesis produce better mechanical responses than the TIS (2-piece) implant when connected to an endodontically treated tooth restored with a post core and crown.

## Background

Systematic reviews to assess the survival and complication rates of fixed partial dentures (FPDs) indicated that the survival rates for both implants and reconstructions combined with tooth implant-supported prosthesis (TISP) were lower than those reported for implant-only supported prosthesis [1–5]. Historically, connecting a tooth to an implant is discouraged because differences in tooth and implant mobility would result in the prosthesis being cantilevered to stress the implant [2–5]. However, splinting the implant and natural tooth is occasionally used as a rational alternative for anatomical structural reasons or patient-centered preferences [1–5]. Endodontically treated tooth restored used a post and core build-up and full crown is occasionally regarded as an abutment tooth in a TISP with healthy periodontal stability and in dense bone situations [4, 6–12]. Potential physiological and engineering problems, such as marginal bone loss and implant or prosthesis complications (fracture) can occur under long-term higher bending moment acting at the implant site owing to the biomechanical dilemma resulting from the dissimilar mobility between an osseointegrated implant and tooth [2, 12–16]. However, the literature contains limited and conflicting data that addresses inclusion of abutment teeth with endodontic therapy in a TISP [4]. A computer simulation study indicated that a 3-piece implant with a retaining-screw (RS) connection system produced higher stress compared with a 2-piece implant with a taper integrated screw-in (TIS) connection system [12]. Unfortunately, the result from this static numerical study requires further evaluation using insight into the in vitro fatigue tests to provide more realistic consultation.

The acoustic emission (AE) technique is a non-destructive technique that offers the advantages of being a non-stop technique that monitors the condition of materials under investigation throughout the test [17, 18]. Combined with conventional static fracture test machines, AE can identify failure initiation, the initial damage site, damage propagation and catastrophic material failure and help elucidate the complex failure mechanism [19–26]. However, the AE technique has rarely been employed in dental biomechanical testing under cyclic loads. Studies successfully combined the AE technique with fatigue shear testing to investigate micro-crack growth and damage in ceramic/tooth tissue adhesive interfaces and found that the cumulative number of AE hits increased with a lower load level in cyclic load tests before fracture [27, 28].

Accordingly, this study applied the AE technique to monitor the resistance in an endodontically treated TISP using different implant macrostructures with 2-(TIS) and 3-(RE) piece implant systems under dynamic cyclic loads. AE signal results at different cyclic load stages were obtained and section-images of the tested samples were evaluated after fatigue testing to understand the biomechanical response in an endodontically treated TISP.

## Methods

### Implant static failure test

**Fig. 1:**
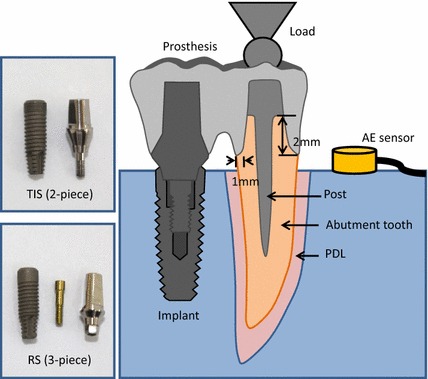
Schematic of the endodontically treated TISP sample including a premolar, artificial PDL, splinting to TIS (2-piece) or RS (3-piece) implant and prosthesis

A 2-piece implant with a TIS connection and a 3-piece implant with an RS connection were selected as the implant systems for investigation (Fig. 1). These two different macrostructure implant systems with the same size (4.5 mm in diameter and 13 mm in length) belonged to the same brand (IDEOSS Ltd. Inc., Taipei, Taiwan) to avoid manufacturing variations.

Three implants of each TIS and RS connection types were used to evaluate the mechanical resistance using the static failure test in conformity with the ISO 14801 standard [29]. All implants were embedded in an epoxy resin block at a distance 3.0 mm apically from the nominal bone level as the bone-anchoring part for all test specimens. The specimen was then clamped at a 30° angle (considered axial and lateral loads) between the implant axis and load direction in a universal testing machine (E3000, Instron, Canton, MA, USA) to perform the failure test. Compressive load was applied through a loading device (cap) at a rate of 1 mm/min until the force decreased below 20 % of the maximum load or the implant failed. The maximum static test force was determined.

### Endodontically treated TISP fatigue test

Ten freshly extracted intact mandibular second premolars with similar size controlling for a maximum deviation of 20 % from the root length and crown dimensions were selected as abutment teeth. The artificial PDL of each premolar was replicated with approximated 0.5 mm thickness around tooth from 1.5 mm below the CEJ to the root using silicon (Gingifast Elastic, Zhermack SpA, Badia Polesine, Italy). Ten abutment teeth and other prepared implants (three implants in each of TIS and RS connection types) were aligned and embedded into an epoxy resin block. The abutment teeth were prepared with a 1 mm wide chamfer finish line and 2 mm ferrule length above the CEJ. The N–Cr alloy post-core was restored and cemented after performing conventional endodontic steps. After wax-up and casting procedures, a Ni–Cr alloy (VeraBond II, Dentech Dental Ltd. London, UK) with two-unit crowns for the premolar and first molar were fabricated and cemented onto the endodontically treated teeth (Fig. 1).

**Fig. 2:**
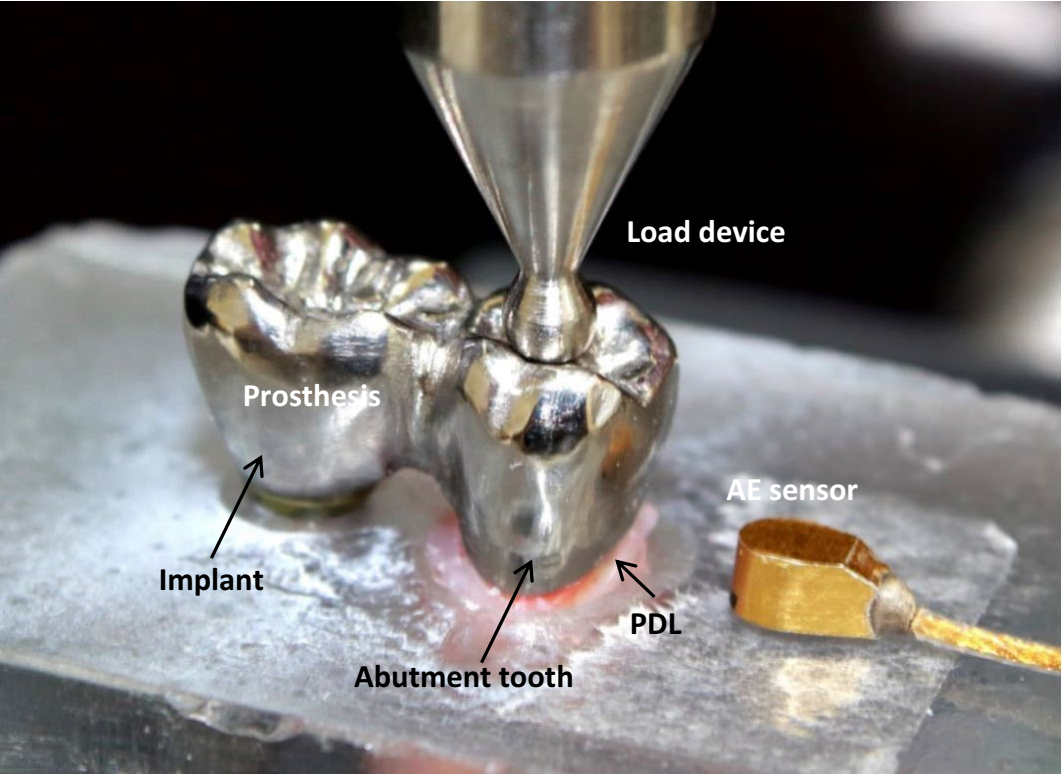
One of the tested endodontically treated TISP samples included a stainless steel sphere in contact with the buccal and lingual cusps for axial load and an AE signal wide band transducer glued with resin to the sample embedded in a *resin block*

The endodontically treated TISP samples were clamped into the INSTRON testing machine to perform the fatigue test. The fatigue test cyclic loads were carried out by applying 20N (Fmin) to 200N (Fmax) onto the abutment tooth with a 4-mm steel sphere contacting the buccal and lingual cusps to simulate the occlusal forces, i.e. the R value (Fmax/Fmin) was set at 10 [30, 31] (Fig. 2). The test frequency was set at 4 Hz because the human mastication frequency was found to be 1 to 4 Hz from the literature [32, 33]. The number of cycles at each load was set at 10^5^ because this number simulated chewing and swallowing for one half year [34, 35]. All samples were stained, subsequently embedded in epoxy resin and bi-sectioned through the implant axis using a low speed diamond saw with copious cooling (CL50 Precision Saw, Top Tech Machines Co., Ltd., Taipei, Taiwan). A non-contact video measurement system (SVP-2010, ARCS Co., Ltd., Taichung, Taiwan) was applied to evaluate endodontically treated TISP detail defects. The images were obtained using 26 times magnification with a color CCD camera and transferred into an imaging program to allow evaluation.

### AE analysis

An AE signal wide band transducer (Broadband sensor S9225, Physical Acoustic Corporation (PAC) Princeton Junction, NJ, USA) was glued with resin (Triad Gel, Dentsply, York, PA, USA) to the resin block for fatigue tests (Figs. 1, 2). The signals acquired with the sensor amplified by a preamplifier with 20/40/60 dB gains was added to the A processing section. The parameters selected for the signal acquisition were: a 40 dB gain for the preamplifier, a 100–2 MHz band pass and a 45 dB threshold [19, 20, 36]. The AE signals were recorded during the load period. The numbers of cycles versus AE hits in the cyclic load test for each sample were recorded to evaluate how the sample failure process progressed. AE signal mean and standard deviations accumulated at each recorded time were computed. All data were statistically analyzed using the *t* test method (α = 0.05).

## Results

The mean maximum load of the static test for RS (3-piece) and TIS (2-piece) implants were 797 ± 13 and 559N ± 26N (mean ± standard deviation), respectively. The statistical t test result of the maximum load displayed significant differences (p < 0.05) between RS and TIS implants. No fracture patterns were found in all test implants but large deformations were indicated at the abutment screw of both the RS and TIS implants after static testing.

**Fig. 3:**
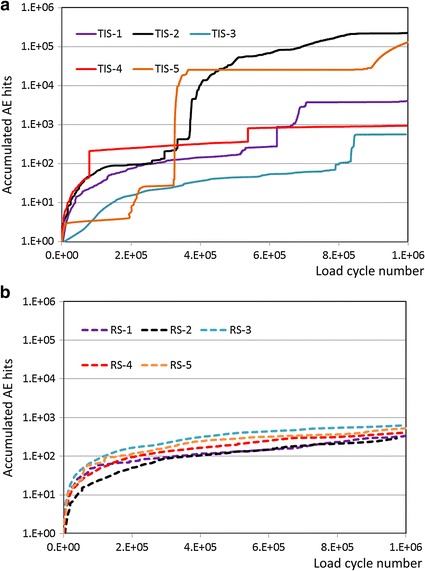
Accumulated number of AE signals versus number of cyclic loads: **a** TIS implant; **b** RS implant

**Fig. 4:**
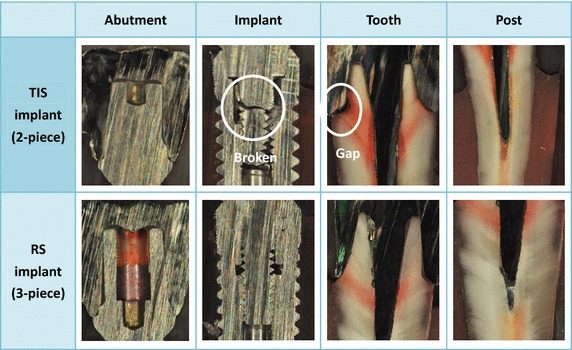
One of the failure modes on behalf of endodontically treated TISP samples using RS and TIS implants, abutment screw fracture was found in the TIS implant. The *color stained image* indicated that a small gap was generated between the crown and tooth in the chamfer in the TIS samples

After 10^5^ cyclic loads, the number of AE signals increased with the cycle load number and accumulated mean number of AE signals for the TIS implant (72511) were much higher than that for the RS implant (437) (Fig. 3; Table 1). Large standard deviation was found in the TIS implant and non-significant differences (p > 0.05) were found in the accumulated number of AE signals between the TIS and RS implants. Unstable TIS implant curves were found in the AE accumulated number versus load cycle diagram (Fig. 3). No obvious damage was noted in endodontically treated TISP samples using RS implants but two of the corresponding TIS implant samples were broken (Table 1). One of the defect evaluation images on behalf of endodontically treated TISP samples using RS and TIS implants are shown in Fig. 4. Abutment screw fracture was found in the TIS implant. The color stained image also indicated that a small gap was generated between the crown and tooth in the chamfer in the TIS samples.

**Table 1:** Number of accumulated AE signals, mean value and implant failure mode under fatigue test of all endodontically treated TISP samples

	TIS (2-piece) implant					RS (3-piece) implant				
Number of accumulated AE signals	4084	565	940	226134	130834	335	286	412	628	522
Mean ± SD	1863 ± 1578					437 ± 125				
Implant failure	Abutment screw fracture	No damage was noted	Abutment screw fracture	Abutment screw fracture	Abutment screw fracture	No damage was noted	No damage was noted	No damage was noted	No damage was noted	No damage was noted

## Discussion

In the TISP, the amount of abutment tooth movement with healthy periodontal ligaments against that of an osseointegrated dental implant can be 5–20 times greater and can produce a series of physiological and engineering complications [1–5]. Although TISP used with a rigid connection has been advocated by some authors and found to reduce the risk of abutment-tooth intrusion. However, other influential factors such as implant type should be further evaluated, especially when an endodontically treated tooth is used as the abutment tooth in TISP. This study was designed to focus on how to reduce the risk of implant complications in an endodontically treated TISP. Our previous numerical study indicated that RS (3-piece) implant might induce higher stress when compared with the TIS (2-piece) implant. However, the clinical significance of the results from static finite element (FE) analysis is occasionally questionable because FE analysis results are usually dominated by many numerical assumptions, such as PDL non-linear material property, ill-condition element, load and boundary conditions. Particularly, a monotonic load does not accurately represent a clinical situation in which repetitive fatigue loading is characteristic [12].

Repeated load fatigue has been recognized as an important concept rather than a catastrophic event, inducing TISP failure. The AE technique has also been employed in orthopaedic/dental material dynamic fatigue testing. The AE signal can be regarded as having high sensitivity as it registers even the smallest cracks as long as a noise is generated and thus is claimed to provide real time data [19]. Cumulative numbers of AE hits are observed to increase with a lower load level in cyclic load tests before fracture [27]. Fracture resistance and small cracks accruing and growth on the testing samples can be estimated depending on the cumulative number of AE hits. Therefore, this study attempted to combine the AE technique to monitor the failure process in an endodontically treated TISP when connected to different implant systems under dynamic loads. The fatigue test result indicated that non-significant differences were found in the accumulated number of AE signals between the RS (3-piece) and TIS (2-piece) implants due to their high standard deviations. A large number of AE signals were found in the four TIS implant samples because the corresponding abutment screws were fractured, producing large standard deviation in this group. This phenomenon can be interpreted as the unstable broken implant structure induced the AE signal to continue to record until the fatigue test was stopped. The AE monitoring system could not identify what load stage lead to the sample fracture during the fatigue test. However, the mean accumulated number of AE signals in the TIS implant were observed higher than those for the RS implant and implied that more micro-cracks might accumulate and grow in the TIS implant. This result can be verified in the implant static failure test that found maximum load for the RS (3-piece) implant (797N) was higher than that for the TIS (2-piece) implant (559N). The static failure testing ensures that the implant or implant/abutment system is subjected to both compressive and shear (lateral) forces, with no lateral constraint occurring. The fracture pattern also evidenced that endodontically treated TISP connected with TIS (2-piece) implant has lower resistance and two of the corresponding samples were broken. This phenomenon indicated that the RS (3-piece) implant might present better mechanical compensation among all components within the implant when receiving the moment condition. The color stained image indicated that a small gap occurred between the crown and tooth in the chamfer in the TIS samples due to the stress concentration generated after the implant broke. These results imply that using the RS (3-piece) implant may be a better option when an endodontically treated tooth is connected to an implant for patients with less than ideal implant sites.

This in vitro experimental study was designed to understand the relationship between the accumulated number of AE signals and fracture (load) resistance in different implant type connection for endodontically treated TISP. Some clinical situations cannot be fully represented in this study and need to be assumed, such as silicon material was only used to simulate the artificial PDL and complex (lateral) occlusal load conditions was not performed in the fatigue test. The axial load applied on the abutment tooth in this study simulated the bending moment effect on the implant. Standard internal implant-abutment connections, such as 6-point internal hex of passive fit (RS connection) and Morse taper with friction fit (TIS connection) were discussed but external butt-joint connection was not considered in this study. Otherwise, the situation regarding the number of occlusal contacts that occur in vivo, Delong et al. [34] addressed that chewing tooth contacts equal 240,000 in 1 year. Ruse et al. [37] pointed out that chewing and swallowing contacts equal 1800 per day. These numbers translate into 10^5^ in 2–5 months. Owing to the constrained time available for collecting data, a 10^5^ cyclic load was used as the relative number to simulate clinical chewing. Therefore, the experimental results provided in this study must be confirmed in further well-controlled long-term clinical trials.
